# Advancing the Concept of Resilience for Older People Who Are Experiencing Homelessness

**DOI:** 10.1093/geroni/igaa057.2487

**Published:** 2020-12-16

**Authors:** Mineko Wada, Sarah Canham, Mei Lan Fang

**Affiliations:** 1 STAR Institute, Vancouver, British Columbia, Canada; 2 University of Utah, Salt Lake City, Utah, United States; 3 University of Dundee, Dundee, Scotland, United Kingdom

## Abstract

Current conceptualizations of resilience have overlooked the lived expertise of older people experiencing homelessness (OPEH) – individuals who have much insight to offer in terms of progressing notions on how people ‘stand up’ to adversity and ‘bounce back’ to a state of physical and psychological homeostasis across the life course. Drawing from extant literature and data from a community-engaged research project, which interviewed 40 participants and examined the health supports needed for individuals experiencing homelessness upon hospital discharge, we provide a comparison of resilience among homeless individuals generally and resilience among OPEH. Based on narratives of significant adversity experienced by OPEH in Vancouver, Canada, we offer a critical analysis of ‘resilience in ecological context’ that identifies unique characteristics of resilience at micro, meso, exo, and macro system levels. We discuss how our conceptual model of resilience pertinent to OPEH can be used to shape research, policy, and practice. Part of a symposium sponsored by the Environmental Gerontology Interest Group.

